# An outbreak of *bla*_OXA-51-like_- and *bla*_OXA-66_-positive *Acinetobacter baumannii* ST208 in the emergency intensive care unit

**DOI:** 10.1099/jmm.0.077503-0

**Published:** 2014-11

**Authors:** Satomi Asai, Kazuo Umezawa, Hideo Iwashita, Toshio Ohshima, Maya Ohashi, Mika Sasaki, Hideki Hayashi, Mari Matsui, Keigo Shibayama, Sadaki Inokuchi, Hayato Miyachi

**Affiliations:** 1 Department of Laboratory Medicine, Tokai University School of Medicine, 143 Shimokasuya, Isehara 259-1193, Japan; 2 Infection Control Division, Tokai University Hospital, 143 Shimokasuya, Isehara 259-1193, Japan; 3 Department of Critical Care and Emergency Medicine, Tokai University School of Medicine, 143 Shimokasuya, Isehara 259-1193, Japan; 4 Support Center for Medical Research and Education, Tokai University School of Medicine, 143 Shimokasuya, Isehara 259-1193, Japan; 5 Department of Bacteriology II, National Institute of Infectious Diseases, Tokyo, Japan

## Abstract

A series of clinical isolates of drug-resistant (DR) *Acinetobacter baumannii* with diverse drug susceptibility was detected from eight patients in the emergency intensive care unit of Tokai University Hospital. The initial isolate was obtained in March 2010 (*A. baumannii* Tokai strain 1); subsequently, seven isolates were obtained from patients (*A. baumannii* Tokai strains 2–8) and one isolate was obtained from an air-fluidized bed used by five of the patients during the 3 months from August to November 2011. The isolates were classified into three types of antimicrobial drug resistance patterns (RRR, SRR and SSR) according to their susceptibility (S) or resistance (R) to imipenem, amikacin and ciprofloxacin, respectively. Genotyping of these isolates by multilocus sequence typing revealed one sequence type, ST208, whilst that by a DiversiLab analysis revealed two subtypes. All the isolates were positive for *bla*_OXA-51-like_ and *bla*_OXA-66_, as assessed by PCR and DNA sequencing. *A. baumannii* Tokai strains 1–8 and 10 (RRR, SRR and SSR) had quinolone resistance-associated mutations in *gyrA/parC*, as revealed by DNA sequencing. The IS*Aba1* upstream of *bla*_OXA-51-like_ and aminoglycoside resistance-associated gene, *armA*, were detected in *A. baumannii* Tokai strains 1–7 and 10 (RRR and SRR) as assessed by PCR. Among the genes encoding resistance–nodulation–division family pumps (*adeB, adeG* and *adeJ*) and outer-membrane porins (*oprD* and *carO*), overexpression of *adeB* and *adeJ* and suppression of *oprD* and *carO* were seen in isolates of *A. baumannii* Tokai strain 2 (RRR), as assessed by real-time PCR. Thus, the molecular characterization of a series of isolates of DR *A. baumannii* revealed the outbreak of ST208 and diverse antimicrobial drug susceptibilities, which almost correlated with differential gene alterations responsible for each type of drug resistance.

## Introduction

*Acinetobacter baumannii* is emerging as a nosocomial pathogen, particularly in intensive care units, including burn care units (Bayram *et al.*, 2013; Guzek *et al.*, 2013; Ohashi *et al.*, 2013). Hospitalized patients at a greater risk of *Acinetobacter* infections are those particularly ill on a ventilator, those with a prolonged hospital stay, those who have open wounds and those with invasive devices, such as urinary catheters (Wendt *et al.*, 1997; Wisplinghoff *et al.*, 2000; Ho *et al.*, 2010; Howard *et al.*, 2012; Zheng *et al.*, 2013).

A stepwise evolution in the acquisition of multidrug resistance in clinical isolates of *Pseudomonas aeruginosa* and *A. baumannii* has been reported (Higgins *et al.*, 2010; Asai *et al.*, 2011). Elucidation of the mechanism(s) underlying the drug resistance is important to prevent such resistance.

We identified a series of clinical isolates of drug-resistant (DR) *A. baumannii* with a diverse pattern of drug resistance from eight patients in the emergency intensive care unit (EICU) of Tokai University Hospital from March 2010 to November 2011. In order to elucidate the diversity on drug resistance in the same sequence type, we studied the molecular characteristics of the clinical isolates and the relationship with the resistance pattern.

## Methods

### 

#### Patients and clinical specimens.

The patients studied were admitted to the EICU (57 beds, including three beds in the severe burn care unit) of Tokai University Hospital (total 804 beds), because of serious burns, traffic injuries or cerebral haemorrhage. During this period, all patients, except two with cerebral haemorrhage, were treated by the systemic administration of antibiotics, including β-lactams, fluoroquinolones, aminoglycosides and glycopeptides, for infections of wounds and/or the respiratory tract.

Routine microbial examinations were performed on a weekly basis on clinical specimens from the patients’ sputum, urine (via catheter), venous blood, wounds, etc.

Bacteriological surveillance of environmental surfaces was performed for the medical equipment shared by the patients in order to seek a possible reservoir of the pathogen, such as an air-fluidized bed. A drug-sensitive strain of *A. baumannii* (*A. baumannii* Tokai strain 9; resistance pattern SSS, see below) was used as a control. The epidemiological investigation was performed to elucidate the possible transmission route using information on the clinical care of the patients and detection of *A. baumannii*. The study was approved by the Review Board of Tokai University (13R-036).

#### Growth conditions and antibiotic susceptibility testing.

Bacteria were cultured at 37 °C in Luria–Bertani broth (Kyokuto Pharmaceutical Industrial). The criteria for multidrug-resistant (MDR) *A. baumannii* was resistance to imipenem (IPM; MIC >16 µg ml^−1^), amikacin (AMK; MIC >32 µg ml^−1^) and ciprofloxacin (CPFX; MIC >4 µg ml^−1^), and DR *A. baumannii* was defined as being resistant to one or two of the drugs according to the Japanese National Guideline Concerning the Prevention of Infections and Medical Care for Patients with Infections.

Biochemical identification and susceptibility testing of the isolates was performed to obtain MIC values using a microdilution method (CLSI, 2009) and the MicroScan WalkAway-96 SI system (Siemens Japan). The antibiotics used in this study were obtained as follows: AMK and IPM were from Banyu Pharmaceutical, aztreonam (AZT) was from Eizai, ceftazidime (CAZ) was from Glaxo SmithKline, cefepime (CFPM) was from Bristol-Myers Squibb, CPFX was from Bayer HealthCare, cefozopran (CZOP) was from Takeda Pharmaceutical, doripenem (DRPM) was from Shionogi, fosfomycin (FOM) was from Meiji Seika, gentamicin (GM) was from Schering-Plough, levofloxacin (LVFX) was from Daiichi-Sankyo Pharmaceutical, meropenem (MEPM) was from Daiichi-Sumitomo Pharmaceutical, minocycline (MINO) was from Wyeth & Takeda Pharmaceutical, piperacillin (PIPC) was from Taisho Toyama Pharmaceutical and tobramycin (TOB) was from Towa Pharmaceutical.

#### Molecular typing.

DNA templates were extracted using a ZR-Duet DNA/RNA MiniPrep kit (Zymo Research). Multilocus sequence typing (MLST) was performed as described previously (Bartual *et al.*, 2005; Fu *et al.*, 2010). MLST sequences were uploaded into the *A. baumannii* MLST Sequence Type Database (http://pubmlst.org/abaumannii/) to determine the alleles and sequence types. *A. baumannii* isolates were screened for gene homology by a repetitive-element-based PCR (rep-PCR) DiversiLab Microbial Typing System (Sysmex bioMérieux), which amplified the regions between the non-coding repetitive sequences in bacterial genomes, as described previously (Carretto *et al.*, 2008; Higgins *et al.*, 2012). The annealing temperature of the PCR amplification used in this study was 55 °C for *gltA*, *gyrB*, *recA* and *cpn60*, and 50 °C for *gdhB*, *gpi* and *rpoD*. The amplification products were purified with a DNA purification kit (Qiagen). The DNA sequencing was performed using an ABI3500xL Genetic Analyzer (Applied Biosystems).

### Evaluation of the mechanisms of resistance

#### Screening for metallo-β-lactamase (MBL).

*A. baumannii* isolates were screened for the production of MBL by a double-disc synergy test with discs containing sodium mercaptoacetic acid as described previously (Arakawa *et al.*, 2000).

#### PCR assay for β-lactamase and armA.

The following resistance genes were examined by PCR: *bla*_IMP-1_, *bla*_VIM_, *bla*_OXA-23-like_, *bla*_OXA-24-like_, *bla*_OXA-51-like_, *bla*_OXA-58-like_ and IS*Aba1*, as described previously (Turton *et al.*, 2006; Woodford *et al.*, 2006). The *armA* gene, which encodes 16S rRNA methylases and confers high resistance to aminoglycosides, was screened by PCR using primers that were described previously (Yamane *et al.*, 2005).

#### Sequencing of OXA-type β-lactamase, and *gyrA* and *parC*.

Sequencing of OXA-type β-lactamase was performed as described previously (Endo *et al.*, 2012). The quinolone resistance-determining regions of *gyrA* and *parC* were amplified and analysed as described previously (Liu *et al.*, 2012). DNA sequencing of the amplified DNA products was performed using an ABI3500xL Genetic Analyzer (Applied Biosystems).

#### Quantitative real-time (qRT)-PCR).

RNA templates were extracted by a ZR-Duet DNA/RNA MiniPrep kit (Zymo Research). The expression levels of three different genes encoding resistance–nodulation–division (RND) family pumps (*adeB*, *adeG* and *adeJ*) and two different genes encoding outer-membrane porins (*oprD* and *carO*) were analysed by qRT-PCR using a StepOnePlus Real-Time PCR System (Applied Biosystems) (Peleg *et al.*, 2008; Fernando & Kumar, 2012; Zander *et al.*, 2013). The primers used for the analysis are listed in Table 1. The housekeeping gene 16S rRNA was used as a control (Coyne *et al.*, 2010; Srinivasan *et al.*, 2011; Hou *et al.*, 2012). Reactions (20 µl) were set up using 400 nM primers and 2 µl cDNA template (diluted 1 : 10) with SYBR Premix Ex *Taq* II (Tli RNaseH Plus) and ROX plus (Takara Bio). The data analysis was carried out using StepOne software. The expression of each target gene was normalized based on the level of the 16S rRNA mRNA gene and was expressed as a relative rate compared with that in the susceptible isolate of each pair (the expression of *A. baumannii* Tokai strain 9 was taken as 1.0). Experiments were conducted at least three times independently and all reactions were carried out in triplicate.

**Table 1.** Primer sequences for qRT-PCR

Primer	Direction	Primer sequence (5'→3′)	Product size (bp)	Reference
*adeB*	Forward	aatactgccgccaataccag	106	Fernando & Kumar (2012)
	Reverse	ggattatggcgactgaagga		
*adeG*	Forward	atcgcgtagtcaccagaacc	92	Fernando & Kumar (2012)
	Reverse	cgtaactatgcggtgctcaa		
*adeJ*	Forward	catcggctgaaacagttgaa	109	Fernando & Kumar (2012)
	Reverse	gcctgaccattaccagcact		
*oprD*	Forward	ccagctcagttgctcaatca	134	This study
	Reverse	catttggtttccagcgtttt		
*carO*	Forward	ggttataacggcggtgacat	115	This study
	Reverse	ccaaggacgaatttcagcat		
16S rRNA	Forward	cgtaagggccatgatgactt	150	Fernando & Kumar (2012)
	Reverse	cagctcgtgtcgtgagatgg		

## Results

### Bacterial strains and antibiotic susceptibility

The characteristics of the *A. baumannii* Tokai strains are shown in Table 2. In March 2010, a DR *A. baumannii* Tokai strain 1 was detected initially from the wound of a patient with a severe burn injury. After 1.5 years, during a period of 3 months from August to November 2011, another seven clinical isolates of DR *A. baumannii* strains from patients (*A. baumannii* Tokai strains 2–8) were obtained. The DR *A. baumannii* Tokai strains were classified into three types according to their susceptibility to three drugs (IPM, AMK and CPFX) as RRR, SRR or SSR (R, resistant; S, susceptible; Tables 2 and 3). They were obtained from sputum, wounds and bile drains.

**Table 2.** Cases and *A. baumannii* Tokai strains One hundred and fifty nurses and five nurse-aids worked in the EICU and Burn centre, and they were not fixed as a team. ER, Critical care and emergency medicine; NR, neurosurgery; OP, orthopaedics; R, resistant; S, susceptible.

Strain/disease	Ward	Day detected after hospitalization	Source	Susceptibility pattern of IPM, AMK and CPFX*	Doctor team	Use of air-fluidized bed
1. 74 % total body surface area burn	Burn centre	31 (3 March 2010)	Sputum	IPM-S, AMK-R, CPFX-R (SRR)	ER-a	Yes
2. 85 % total body surface area burn (Ohashi *et al.*, 2013)	Burn centre	9 (29 August 2011)	Wound	IPM-R, AMK-R, CPFX-R (RRR)	ER-b	Yes
3. 40 % total body surface area burn (Ohashi *et al.*, 2013)	Burn centre	33 (19 September 2011)	Wound	IPM-R, AMK-R, CPFX-R (RRR)	ER-c	No
4. 70.5 % total body surface area burn (Ohashi *et al.*, 2013)	Burn centre	7 (19 September 2011)	Wound	IPM-R, AMK-R, CPFX-R (RRR)	ER-c	Yes
5. Traffic injury	EICU	44 (23 September 2011)	Bile drain	IPM-S, AMK-R, CPFX-R (SRR)	ER-d	Yes
6. Traffic injury	EICU	13 (26 October 2011)	Wound	IPM-R, AMK-R, CPFX-R (RRR)	ER-d	Yes
7. Iliopsoas muscle abscess	EICU	45 (15 November 2011)	Sputum	IPM-S, AMK-R, CPFX-R (SRR)	OP	No
8. Subcortical haemorrhage	EICU	240 (15 November 2011)	Sputum	IPM-S, AMK-S, CPFX-R (SSR)	ER-d	No
9. Subarachnoid haemorrhage	EICU	50 (17 November 2011)	Sputum	IPM-S, AMK-S, CPFX-S (SSS)	NS	No
10. Air-fluidized bed	EICU	(20 November 2011)	Beads	IPM-S, AMK-R, CPFX-R (SRR)	ER-a, ER-b, ER-c, ER-d	

**Table 3.** Susceptibility patterns of *A. baumannii* Tokai strains

Strain	MIC (µg ml^−1^)															
	β-Lactams								Aminoglycosides			Fluoroquinolones		Other agents		
	IPM	PIPC	CAZ	CFPM	S/C	AZT	MEPM	CZOP	GM	TOB	AMK	LVFX	CPFX	MINO	FOM	S/T
1, 5, 7, 10	**2**	>64	>16	16	<16	16	>8	16	>8	>8	**>32**	4	**>2**	4	>16	>2
2, 3, 4, 6	**>8**	>64	>16	16	<16	16	>8	16	>8	>8	**>32**	>4	**>2**	≤2	>16	>2
8	≤**1**	≤8	≤2	<4	<16	8	≤1	4	≤1	≤1	≤**4**	>4	**>2**	≤2	>16	≤2
9	≤**1**	≤8	4	16	<16	8	≤1	8	8	2	**8**	≤0.5	**1**	≤2	>16	≤2

S/C, sulbactam/cefoperazone; S/T, sulfamethoxazole/trimethoprim.

As the interval between the first patient and the others was long (>18 months), the environment of the ward was suspected to be a possible reservoir of the pathogen. Based on the results of the bacteriological surveillance of environmental surfaces, *A. baumannii* Tokai strain 10 was isolated from the cracks of a rubber frame and a lump of beads in an air-fluidized bed that was used by five patients during their hospitalization (*A. baumannii* Tokai strains 1, 2 and 4–6).

### Molecular typing

**Fig. 1.**
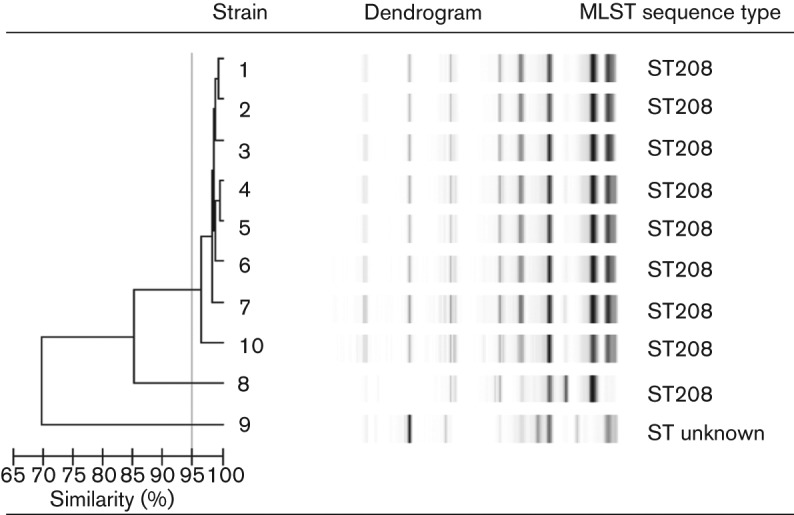
Results of the rep-PCR analysis and MLST in clinical isolates of *A. baumannii*. *A. baumannii* Tokai strains 1–7 and 10 showed identical patterns and homologous rates of identity >97 %. Two isolates, *A. baumannii* Tokai strains 8 and 9, had different patterns (85 and <70 % similarity, respectively). MLST analysis revealed that *A. baumannii* Tokai strains 1–8 and 10 were of the same sequence type (ST208).

The molecular genotyping of isolates by a MLST analysis revealed a sequence type of ST208 for *A. baumannii* Tokai strains 1–8 and 10 (ST profile, *gltA*-*gyrB*-*gdhB*-*recA*-*cpn60*-*gpi*-*rpoD*: 1-3-3-2-2-97-3) and another type for *A. baumannii* Tokai strain 9 (ST profile, *gltA*-*gyrB*-*gdhB*-*recA*-*cpn60*-*gpi*-*rpoD*: 15-48-58-42-36-54-41). The molecular genotyping of isolates by rep-PCR showed the same pattern (>97 % similarity) as one type for eight of the isolates (*A. baumannii* Tokai strains 1–7 and 10) (Fig. 1), and the other isolates (*A. baumannii* Tokai strains 8 and 9) had different patterns (85 and <70 % similarity, respectively).

### Expression of resistance-related genes

The MBL assay of the clinically isolated *A. baumannii* Tokai strains revealed no apparent MBL production and all isolates showed expression of OXA-51-like carrying OXA-66 β-lactamase (Table 4). The expression of IMP-1, VIM, OXA-23-like, OXA-24-like and OXA-58-like was negative. Expression of IS*Aba1* and *armA* was found in *A. baumannii* Tokai strains 1–7 and 10. The DNA sequencing of *gyrA* and *parC* revealed that Ser83 (TCA) was changed to TTA (Leu) and that Ser80 (TCG) was changed to TTT (Phe) or TTG (Leu) in *A. baumannii* Tokai strains 1–8 and 10.

**Table 4.** Expression of resistance-related genes as assessed by PCR and qRT-PCR in *A. baumannii* Tokai strains

Strain(s)	Susceptibility pattern	Gene expression							Mutation	
		OXA-type β-lactamase					IS*Aba1*	*armA*	*gyrA* (Ser83)	*parC* (Ser80)
		OXA-23-like	OXA-24-like	OXA-51-like	OXA-58-like	OXA-66
1, 5, 7, 10	SRR	−	−	+	−	+	+	+	Leu	Leu
2, 3, 4, 6	RRR	−	−	+	−	+	+	+	Leu	Leu
8	SSR	−	−	+	−	+	−	−	Leu	Phe
9	SSS	−	−	+	−	+	−	−	Ser	Ser

Our analysis of genes encoding RND pumps included an analysis of the expression of three previously characterized genes, *adeB*, *adeG* and *adeJ*, which encode the RND pump in the *adeABC*, *adeFGH* and *adeIJK* operons, respectively. The result of *A. baumannii* Tokai strains 1, 2, 8 and 9 as representative strains from each group with the same susceptibility pattern is shown in Table 5. Overexpression of *adeB* and *adeJ* was seen in *A. baumannii* Tokai strain 2. The expression of *oprD* was decreased in *A. baumannii* Tokai strains 2 and 8. Underexpression of *carO* was seen in isolates with *A. baumannii* Tokai strains 1, 2 and 8.

**Table 5.** Relative expression of efflux pumps and outer-membrane porins in *A. baumannii* Tokai strains by qRT-PCR The results for *A. baumannii* Tokai strains 1, 2, 8 and 9 are shown as a representative strain from each group with the same susceptibility pattern.

Strain	Susceptibility pattern	Relative expression				
		Efflux pump (ratio)			Outer-membrane porin	
		*adeB*	*adeG*	*adeJ*	*oprD*	*carO*
1	SRR	0.91	0.46	0.94	1.23	0.02
2	RRR	2.28	1.02	2.41	0.49	0.01
8	SSR	0.10	0.81	0.84	0.88	0.003
9	SSS	1.00	1.00	1.00	1.00	1.00

## Discussion

We investigated a series of clinical isolates of DR *A. baumannii* ST208 in the EICU of Tokai University Hospital. In order to elucidate the diversity of the drug resistance patterns in the same sequence type in these isolates, we studied the molecular characteristics of these isolates and their relationship with the resistance pattern.

*A. baumannii* Tokai strains 1–7 and 10 were positive for OXA-51-like and OXA-66 β-lactamase and IS*Aba1*. *A. baumannii* strains with resistance to AMK (*A. baumannii* Tokai strains 1–7 and 10) were positive for *armA*. These results are consistent with the idea that IS*Aba1* regulates the expression of OXA-51-like carrying OXA-66 β-lactamase and that *armA* is related to aminoglycoside resistance. The five isolates (*A. baumannii* Tokai strains 1, 2 and 4–6) could have been derived from the same source and/or transmitted horizontally, because the same air-fluidized bed had been used by those patients. Among them, *A. baumannii* Tokai strains 2, 4 and 6 showed multidrug resistance (RRR). These patients were treated with carbapenem (MEPM or DRPM) prior to sampling for at least 1 week, which may have played a role in the overexpression of *adeB* and *adeJ* in *A. baumannii* Tokai strain 2. *A. baumannii* Tokai strain 10 was detected from the cracks of the rubber frame and a lump of beads in an air-fluidized bed, even though the bed had been cleaned and disinfected every time after use. Although a few nosocomial outbreaks of *A. baumannii* ST2 have been reported (Suzuki *et al.*, 2013; Yamada & Suwabe, 2013), an outbreak of *A. baumannii* ST208 has not been reported previously in Japan.

As the pattern of the rep-PCR and sequence type of MLST in the eight isolates was the same as that in the initial case, it was suggested that the strain survived for 1.5 years in the environmental reservoir. As infection control procedures, careful attention to environmental cleaning and disinfection in order to reduce the risk of transmission is suggested. *A. baumannii* Tokai strain 8 (SSR) was also ST208, but had a different pattern as shown by rep-PCR. During the transmission from the same original organism, the presence of a transposon or the insertion of a different plasmid might have led to the different pattern. During the period of an outbreak, *A. baumannii* with different drug susceptibility patterns appeared depending on the various resistance mechanisms.

Nine isolates (*A. baumannii* Tokai strains 1–8 and 10) had resistance to CPFX, which can be explained by the mutations of *gyrA* and *parC*. Another major factor contributing to the resistance of this organism was the overexpression of the RND pumps (Fernando & Kumar, 2012; Amin *et al.*, 2013; Zander *et al.*, 2013). Our analysis of genes encoding RND pumps included the expression of three previously characterized genes, *adeB*, *adeG* and *adeJ*, which encode the RND pumps in the *adeABC*, *adeFGH* and *adeIJK* operons, respectively. Efflux pumps such as AdeABC have been reported to be involved in multidrug resistance (Vila *et al.*, 2007; Hou *et al.*, 2012). In our study, *A. baumannii* Tokai strain 2 (RRR) showed overexpression of *adeB* and *adeJ*. *A. baumannii* Tokai strain 8 showed better sensitivity to some β-lactams (CAZ, CFPM and CZOP) than that of *A. baumannii* Tokai strain 9 (SSS). This phenomenon might be associated with underexpression of *adeB*. Two pumps, such as *adeB* and *adeJ*, have been related to the acquisition of multidrug resistance. As for porins, the overexpression of genes encoding RND pumps and the downregulation of genes encoding porins is known to be common in clinical isolates of *Acinetobacter* spp. (Fernando et al., 2013). Our findings also suggest that the underexpression of *carO* in combination with or without *oprD* does not result in resistance to carbapenem in *A. baumannii* Tokai strains 1 and 8 (SRR and SSR). This observation is consistent with previous findings showing that a decrease in porins among *Acinetobacter* strains is not associated with resistance to carbapenems in the presence of β-lactamases (Rumbo *et al.*, 2013; Singh *et al.*, 2013).

In conclusion, we demonstrated that drug resistance is associated with the expression of IS*Aba1* and *armA*, and mutations in *gyrA* and *parC*, and that the overexpression of *adeB* and *adeJ* plays a role in the multidrug resistance of *A. baumannii* Tokai strain ST208.
